# The Effect of Long-Term Exercise on the Production of Osteoclastogenic and Antiosteoclastogenic Cytokines by Peripheral Blood Mononuclear Cells and on Serum Markers of Bone Metabolism

**DOI:** 10.1155/2016/5925380

**Published:** 2016-08-24

**Authors:** J. Kelly Smith, Rhesa Dykes, David S. Chi

**Affiliations:** Departments of Academic Affairs and Biomedical Sciences, James H. Quillen College of Medicine, East Tennessee State University, Johnson City, TN 70571, USA

## Abstract

Although it is recognized that the mechanical stresses associated with physical activity augment bone mineral density and improve bone quality, our understanding of how exercise modulates bone homeostasis at the molecular level is lacking. In a before and after trial involving 43 healthy adults, we measured the effect of six months of supervised exercise training on the spontaneous and phytohemagglutinin-induced production of osteoclastogenic cytokines (interleukin-1*α*, tumor necrosis factor-*α*), antiosteoclastogenic cytokines (transforming growth factor-*β*1 and interleukins 4 and 10), pleiotropic cytokines with variable effects on osteoclastogenesis (interferon-*γ*, interleukin-6), and T cell growth and differentiation factors (interleukins 2 and 12) by peripheral blood mononuclear cells. We also measured lymphocyte phenotypes and serum markers of bone formation (osteocalcin), bone resorption (C-terminal telopeptides of Type I collagen), and bone homeostasis (25 (OH) vitamin D, estradiol, testosterone, parathyroid hormone, and insulin-like growth factor 1). A combination of aerobic, resistance, and flexibility exercises done on average of 2.5 hours a week attenuated the production of osteoclastogenic cytokines and enhanced the production of antiosteoclastogenic cytokines. These changes were accompanied by a 16% reduction in collagen degradation products and a 9.8% increase in osteocalcin levels. We conclude that long-term moderate intensity exercise exerts a favorable effect on bone resorption by changing the balance between blood mononuclear cells producing osteoclastogenic cytokines and those producing antiosteoclastogenic cytokines. This trial is registered with Clinical Trials.gov Identifier: NCT02765945.

## 1. Introduction 

Interest in cytokines as regulators of bone metabolism began with the experiments of Horton and associates who, in 1972, found that conditioned medium from PHA-stimulated peripheral blood mononuclear cells contained bone resorbing activity [1]. This activity was eventually found to be due to interleukin- (IL-) 1 and tumor necrosis factor- (TNF-) *α* [2, 3], prompting a series of studies examining the role of these and other proinflammatory cytokines as mediators of bone resorption in periodontal disease, rheumatoid arthritis, osteolytic malignancies, and osteoporosis [4–8]. In addition to IL-1 and TNF-*α*, the spectrum of cytokines with effects on bone metabolism now includes the antiosteoclastogenic cytokines IL-4 and IL-10 and transforming growth factor- (TGF-) *β*, and two pleiotropic cytokines, interferon- (IFN-) *λ* and IL-6, whose effects on bone vary depending on experimental conditions [9, 10].

Along with fall prevention and calcium and vitamin D supplementation, the Surgeon General has recommended regular physical activity as the first line in fracture prevention in persons with low bone density [11]. Evidence indicates that the mechanical stresses associated with physical activity augment bone mineral density and improve bone quality by promoting adaptive changes in its geometry and architecture [12]. In the microgravity of space, however, bone loss continues at an average rate of one to two percent per month despite the institution of vigorous exercise programs designed to duplicate conditions on Earth [13, 14], indicating that there remains much to be learned about the mechanism(s) whereby physical exercise (and gravity) influences the ontogeny and functioning of hematopoietic and bone cells involved in the maintenance of bone health.

We have investigated the possibility that long-term moderate intensity exercise improves bone health by favorably altering the production of cytokines with osteoclastogenic and antiosteoclastogenic properties by peripheral blood mononuclear cells.

## 2. Methods

### 2.1. Subjects

This before and after clinical study was approved by the Institutional Review Board of East Tennessee State University. Each subject read and signed the informed consent in the presence of an investigator.

**Figure 1:**
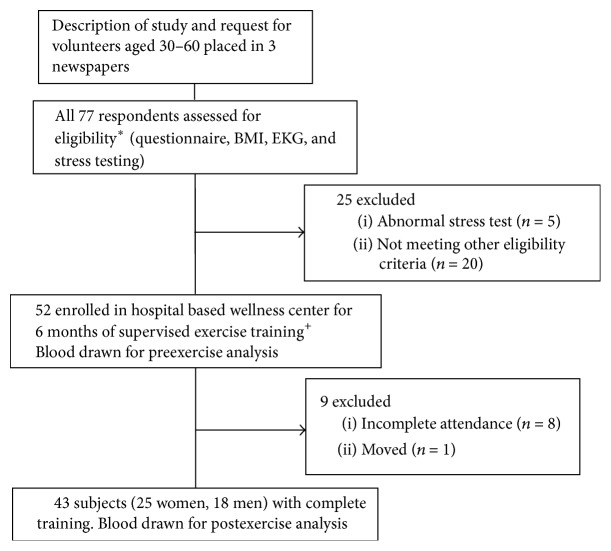
Flow diagram.  ^*∗*^Eligibility criteria: normal EKG, stress test, and exercise tolerance; absence of chronic inflammatory disease, malignancy, immunodeficiency, and immunosuppressive therapy.  ^+^Each training session consisted of a combination of aerobic (60%), resistance (weightlifting) (30%), and flexibility (stretching) (10%) exercises.

Subjects aged 30 to 60 were recruited from the general population by placing an outline of the study and a request for volunteers in three local newspapers. A total of 77 persons responded and all agreed to be screened for eligibility (see Figure 1). Fifty-two volunteers met eligibility criteria and were enrolled in a hospital-based wellness center where they underwent 6 months of supervised training. Subjects were required to exercise for a minimum of 30 minutes twice a week. Each training session consisted of a combination of aerobic (60%), resistance (weightlifting) (30%), and flexibility (stretching) (10%) exercises. Aerobic exercise choices included walking, running, cycling, rowing, climbing, skiing, and aerobics.

Forty-three subjects (25 women, average age 48 years [range 30–58], and 18 men, average age 49 years [range 35–59]) successfully completed the study; their risk factors for osteoporosis are listed in Table 1. Nine subjects were excluded: 8 did not meet attendance requirements and 1 moved to another location.

Supervisors kept detailed records documenting attendance, the duration and type of each exercise, weights, changes in medications, changes in dietary or smoking habits, and state of health. These data, along with risk factors for osteoporosis, were analyzed for their potential effects on outcome measurements (cytokine production, C-terminal telopeptides of Type I collagen, osteocalcin levels, hormonal levels, lymphocyte phenotypes, and mitogen responses) and for group and within-group differences.

Immunologic studies were done at baseline and after completion of 6 months of training. In order to minimize seasonal influences on the results, 2-3 subjects were enrolled each month over an eleven-month period. Subjects were instructed not to exercise outside of the supervised exercise program and for at least 24 hours prior to blood drawing. All blood samples were drawn in the morning at approximately the same time of day.

### 2.2. Serum Assays

Solid-phase enzyme-linked immunoassay kits were used to measure serum levels of osteocalcin (BRI-Diagnostics Bioresearch, Dublin, Ireland), C-terminal telopeptides of Type I collagen (CTXI) (Osteometer Biotech A/S, Herlev, Denmark), and relevant hormones (25 (OH) vitamin D, estradiol, testosterone, parathyroid hormone, and insulin-like growth factor 1) (American Laboratory Products Company, Salem, New Hampshire).

### 2.3. Lymphocyte Phenotyping

Immunophenotyping of blood lymphocytes was done as previously described [52] using lysed whole blood and a FACScan flow cytometer (Becton Dickinson, San Jose, Calif.) and fluorescein- and phycoerythrin-labeled murine monoclonal IgG antibodies to measure levels of T lymphocytes (CD3^+^); T helper lymphocytes (CD4^+^); T cytotoxic lymphocytes (CD8^+^); T lymphocytes displaying MHC class II antigen (DR^+^), vascular adhesion molecule-4 (VLA-4) (CD49d^+^), lymphocyte function-associated antigen-1 (LFA-1) (CD11a^+^), Fas antigen (CD95^+^), or gamma-delta T cell receptors (TCR*γδ* ^+^); B lymphocytes (CD3^−^CD19^+^); B1 lymphocytes (CD3^−^CD19^+^CD5^+^) and natural killer cells (NK cells) (CD3^−^CD16^+^CD56^+^).

### 2.4. Cytokine Production

Cytokine production was measured as previously described [52]. Peripheral blood mononuclear cell (PBMC) preparations containing 15–20% monocytes and 80–85% lymphocytes were isolated from venous blood using Accu-Prep*™* (Accurate Chemical & Scientific Corp., Westbury, NY), washed three times at 10°C with sterile phosphate buffered saline (pH 7.4, 0.1 M), and suspended at a concentration of 2 × 10^6^ cells/*μ*L in RPMI-1640 containing 5% heat inactivated human AB serum (v/v), L-glutamine (2 mM), penicillin (50 U/mL), and gentamicin and streptomycin (50 *μ*g/mL each). Preparations were incubated under 5% CO_2_ at 37°C for 48 hours with and without phytohemagglutinin (PHA) (5 *μ*g/mL), a lectin mitogen that stimulates unprimed T cells to proliferate by cross-linking their T cell receptors (this assay provides a more definitive assessment of T cell function than assays using unstimulated (PHA^−^) cultures). Culture supernatants were rendered cell-free by centrifuging at 1,000 ×g for 10 minutes at 12°C and stored in 1-2 mL aliquots at −80°C for later use. Supernatants were subsequently assayed for IL-1*α*, IL-2, IL-4, IL-6, IL-10, IL-12, TNF-*α*, IFN-*γ*, and TGF-*β*1 using solid-phase enzyme-linked immunoassay kits (Quantikine, R & D Systems, Inc., Minneapolis, MN, for IL-1*α* and TGF-*β*1; Predicta, Genzyme Corp., Cambridge, MA, for TNF-*α*; Cytoscreen, BioSource International, Inc., Camarillo, CA, for IFN-*γ*; and Immunotech, Inc., Westbrook, ME, for IL-2, IL-4, IL-6, IL-10, and IL-12).

### 2.5. Mitogenic Assays

Mitogenic responses were measured by adding methyl-^3^H-thymidine (20 *μ*Ci/mL, 50 *μ*L) to culture samples removed 6 hours prior to supernatant harvesting. Labeled samples were incubated under 5% CO_2_ at 37°C for an additional 6 hours, and the cells were collected on glass fiber filter paper using a Mash II Cell Harvester. Samples were air-dried, placed in vials containing Scinti-Verse II, and assayed with a Beckman LS 9800 liquid scintillation counter. Proliferative responses are expressed as net counts per minute (Δcpm), calculated as cpm in PHA-stimulated cultures minus cpm in unstimulated cultures.

### 2.6. Statistical Analysis

Statistical analysis was done using STATISTICA (Statsoft, Inc., Tulsa, OK). The two-sided* t*-test for dependent samples was used to determine the significance of differences between measurements. The Pearson correlation test was used to quantify the relationship between two variables and linear regression was used to determine the predictability of* Y* from* X*. One-way analysis of variance (ANOVA) was used to compare measurements of three or more groups. Unless stated otherwise, results are expressed as the mean ± (SEM).

## 3. Results

### 3.1. Cytokine Production by PBMCs

In this analysis, we have identified IFN-*γ* as an osteoclastogenic cytokine and IL-6 as an antiosteoclastogenic cytokine. In our opinion, this most accurately reflects their effects on bone formation and resorption. The reader is referred to Table 2 for a summary of the effects of these and the other studied cytokines on bone homeostasis and immune cell function.

### 3.2. Osteoclastogenic Cytokines

**Figure 2:**
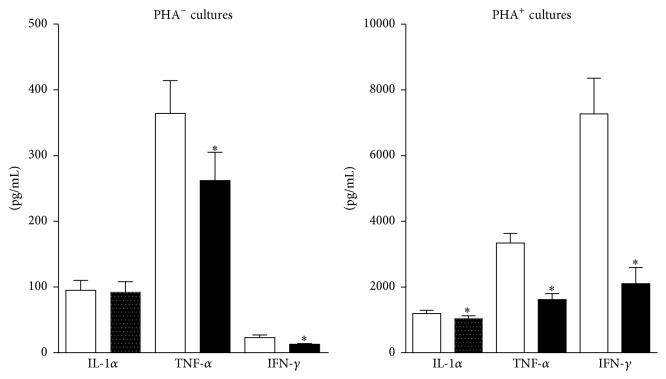
Effect of exercise on osteoclastogenic cytokine production. Exercise attenuated the production of TNF-*α* and IFN-*γ* in both PHA^−^ and PHA^+^ cultures. IL1-*α* values also fell in PHA^+^ cultures. Preexercise values are represented by the white columns and postexercise values by the black columns. Results are given as the mean ± SEM. *∗* indicates a* p* value of <.05 (two-sided* t*-test for dependent samples). PHA: phytohemagglutinin.

Postexercise TNF-*α* levels fell by 52% and 28% in cultures with and without added PHA (*p* ≤ .0031). IL1-*α* levels fell by 13% in PHA^+^ cultures (*p* = .0472) and by 3% in PHA^−^ cultures (*p* > .05). IFN-*γ* levels fell by 71% in PHA^+^ cultures (*p* < .0001) and by 44% in PHA^−^ cultures (*p* = .0065) (Figure 2).

**Figure 3:**
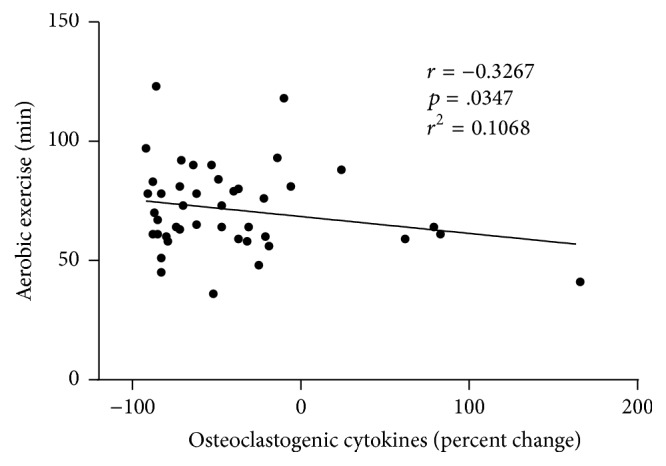
Exercise modality and cytokine production. Osteoclastogenic cytokine levels fell in proportion to the time subjects spent in each training session doing aerobic exercises (Pearson correlation test with regression analysis). Data points for cytokines are the sum of IL-1*α*, TNF-*α*, and IFN-*γ* values.

Collectively, osteoclastogenic cytokine production fell by 59% in PHA^+^ cultures (*p* < .0001) and by 24% in PHA^−^ cultures (*p* > .05); in PHA^+^ cultures, the percent reduction was proportionate to the average time subjects spent per training session doing aerobic exercises (Figure 3).

### 3.3. Antiosteoclastogenic Cytokines

**Figure 4:**
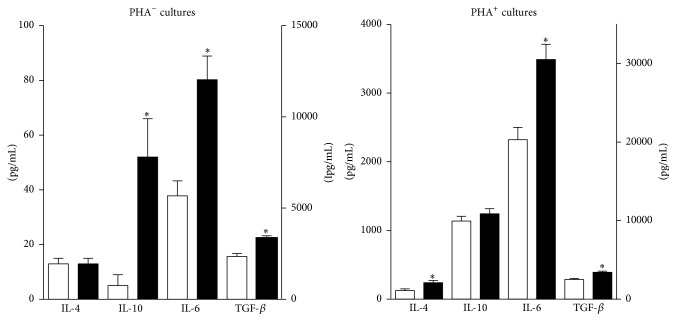
Effect of exercise on antiosteoclastogenic cytokine production. Exercise enhanced the production of IL-6, IL-10, and TGF-*β* in PHA^−^ cultures and IL-4, IL-6, and TGF-beta in PHA^+^ cultures. Preexercise values are represented by the white columns and postexercise values by the black columns. Values for IL-4 and IL-10 are listed on the left* y*-axis, values for IL-6 and TGF-*β*1 are listed on the right* Y* axis. Results are given as the mean ± SEM. *∗* indicates a* p* value of <.05 (two-sided *t*-test for dependent samples). PHA: phytohemagglutinin.

**Figure 5:**
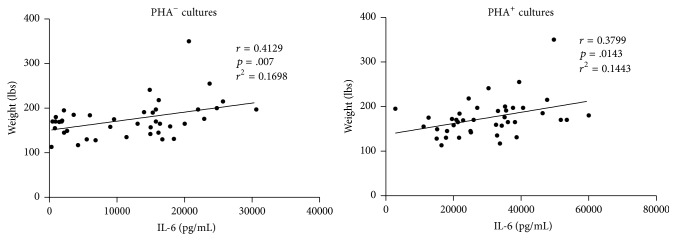
Body weight and IL-6 levels. In both PHA^−^ and PHA^+^ cultures, IL-6 values correlate linearly with body weight (Pearson correlation test with regression analysis).

Postexercise TGF-*β*1 levels increased by 37% and 43% in cultures with and without added PHA (*p* < .0001). IL-10 levels increased by 9% in PHA^+^ cultures (*p* > .05) and by 940% in PHA^−^ cultures (*p* = .0025). IL-4 levels increased by 94% in PHA^+^ cultures (*p* = .0012) and showed no change in PHA^−^ cultures. IL-6 levels increased by 50% in PHA^+^ cultures and by 112% in PHA^−^ cultures (*p* < .0001) (Figure 4). In both PHA^+^ and PHA^−^ cultures, IL-6 production was linearly related to body weight (Figure 5).

Collectively, antiosteoclastogenic cytokine production increased by 50% in PHA^+^ cultures and by 89% in PHA^−^ cultures (*p* < .0001).

### 3.4. Growth and Differentiation Cytokines

**Figure 6:**
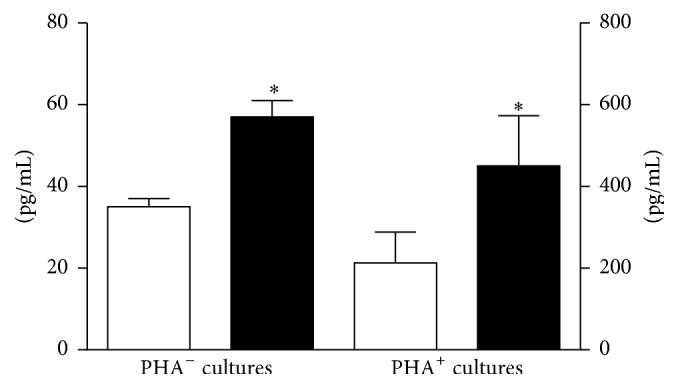
Effect of exercise on IL-2 production. Exercise increased the production of IL-2 in both PHA^−^ and PHA^+^ cultures. Preexercise values are represented by the white columns and postexercise values by the black columns. PHA^−^ culture values are listed on the left* Y* axis and PHA^+^ culture values on the right* Y* axis. Results are given as the mean ± SEM. *∗* indicates a* p* value of <.05. PHA: phytohemagglutinin.

Postexercise IL-2 levels increased by 116% in PHA^+^ cultures (*p* = .0470) and by 63% in PHA^−^ cultures (*p* < .0001) (Figure 6). IL-12 levels fell by 40% in PHA^+^ cultures (*p* > .05) and showed no change in PHA^−^ cultures.

### 3.5. Lymphocyte Phenotypes

Levels of lymphocytes expressing LFA-1, an integrin that augments endothelial cell adhesion, increased by 21% in response to exercise (*p* = .0033). B1 (IL-10^+^ B repressor) cell counts and percentages increased by 330% (*p* = .0580) and 190% (*p* = .0464), respectively, and CD4/CD8 ratios fell by 5.6% (*p* = .0129). There were no significant changes in the levels or percentages of other lymphocyte phenotypes.

### 3.6. Mitogenic Responses

PHA-induced proliferative responses of T cells fell from 9,142 ± 760 Δcpm to 3,155 ± 584 Δcpm following exercise (*p* < .0001). The reason for this change is not known, although it may reflect the change in CD4/CD8 ratios and an increased activity of suppressor cell populations (e.g., M2 monocytes, B1 cells, and T repressor cells).

### 3.7. Serum Factors

**Figure 7:**
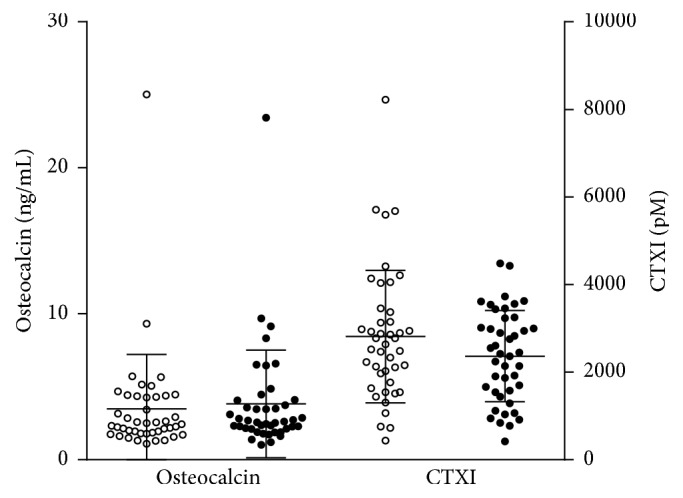
Scattergram showing the effects of the exercise training program on serum levels of osteocalcin and CTXI. Osteocalcin levels increased by 9.8 percent (*p* > .05) and CTXI levels fell by 16% (*p* = .0128). Preexercise values are depicted by white circles and postexercise values by black circles. Osteocalcin levels are shown on the left* Y* axis and CTXI levels on the right* Y* axis. Error bars show the mean ± (SEM) (2-sided* t*-test for dependent samples).

CTXI levels fell by 16% (*p* = .0128) and osteocalcin levels increased by 9.8% (*p* > .05) in response to the exercise program (Figure 7). There was no significant change in serum levels of estradiol, testosterone, parathyroid hormone, or insulin-like growth factor-1 following exercise.

**Figure 8:**
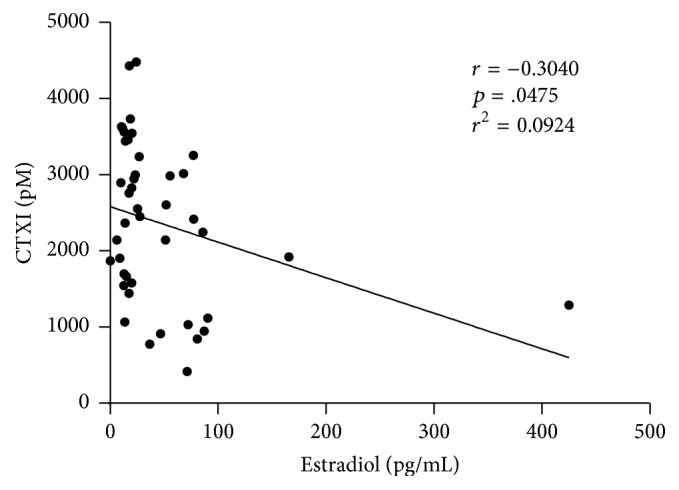
Estradiol and the antiresorptive effect of exercise. Postexercise CTXI levels correlate inversely with estradiol levels (Pearson correlation test with regression analysis). The results suggest that estradiol enhanced the antiresorptive effects of the exercise training program. Note: the correlation improves with removal of the apparent outlier (425 pg/mL estrogen): *r* = −0.3376 and *p* = .029.

Postexercise CTXI levels correlated inversely with estradiol levels (Figure 8).

### 3.8. Exercise Parameters

Subjects trained an average of 2.5 hours per week (range 0.3–7.4 hours). The average duration of each exercise session was 71 minutes (range 36–123 minutes), and the average number of visits per week was 2 (range 1 to 5). During each training session, subjects divided their time between aerobic (57%), resistance (weightlifting) (35%), and flexibility (stretching) (8%) exercises. Aerobic exercises included walking or running (32%), cycling (16%), aerobics (3%), rowing (3%), climbing (2%), and skiing (1%).

### 3.9. Group and Within-Group Variations

#### 3.9.1. Cytokines

PBMCs taken from men prior to exercise spontaneously produced more IFN-*γ* and TNF-*α* than mononuclear cells taken from women (*p* ≤ .04). Following exercise, men spontaneously produced more IL1-*α* and TNF-*α* than women (*p* ≤ .004).

**Figure 9:**
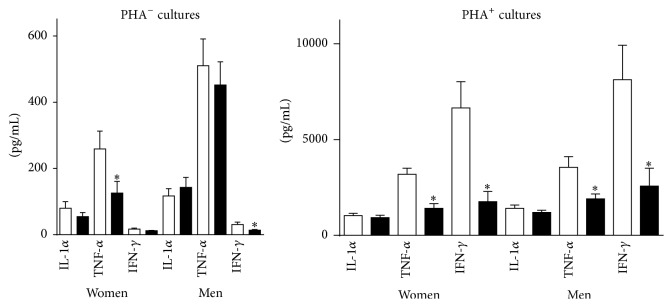
Osteoclastogenic cytokines in women and men. Exercise attenuated the production of TNF-*α* and IFN-*γ* in PHA^+^ cultures in both women and men. In PHA^−^ cultures, levels of TNF-*α* (women) and IFN-*γ* (men) also fell. The white and black columns represent pre- and postexercise levels, respectively. Results are given as the mean ± SEM. *∗* indicates a* p* value of <.05 (two-sided* t*-test for dependent samples). Note that men spontaneously produced more osteogenic cytokines than women both before and after exercise.

**Figure 10:**
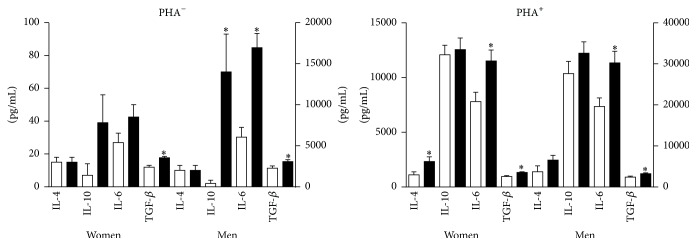
Antiosteoclastogenic cytokines in women and men. In PHA^−^ cultures, exercise enhanced the production of TGF-*β* in women and IL-10, IL-6, and TGF-*β* in men. In PHA^+^ cultures, exercise enhanced the production of IL-4, IL-6, and TGF-*β* in women and IL-6 and TGF-*β* in men. The white and black columns represent pre- and postexercise levels, respectively. In both figures, IL-4 and IL-10 values are listed on the left* Y* axis and IL-6 and TGF-*β* values on the right* Y* axis. Results are given as the mean ± SEM. *∗* indicates a* p* value of <.05 (two-sided* t*-test for dependent samples). Note that, in PHA^−^ cultures, postexercise production of IL-10 and IL-6 is greater in men than in women.

In both men and women, exercise attenuated the production of TNF-*α* and IFN-*γ* (*p* ≤ .0062) and enhanced the production of IL-6 and TGF-*β*1 (*p* ≤ .0028) in PHA-stimulated cultures. Postexercise levels of TGF-*β* also increased in PHA^−^ cultures in both groups (*p* ≤ .0049) (two-sided* t*-test for dependent samples) (Figures 9 and 10).

#### 3.9.2. Habits and Body Metrics

Four subjects (9.3%) changed their diet to one that was lower in energy intake and animal fat, and 2 of the 5 tobacco users discontinued smoking during the study. There were no changes in medications or alcohol consumption.

By completion of the study, 32% of the women and 27% of the men had lost weight, and 4% of the women and the 11% of men had gained weight; there was no significant change in the mean weight of either group.

No group or within-group differences could be demonstrated as a result of weight change, menopause, use of medications, alcohol consumption, or smoking (one-way ANOVA).

## 4. Discussion

The primary effect of our exercise program was to change the balance between PBMCs producing osteoclastogenic cytokines and those producing antiosteoclastogenic cytokines. It is likely that similar changes occurred in hematopoietic cells occupying the microenvironment of bone where they are ideally situated to influence the ontogeny and functioning of cells responsible for bone formation (osteoblasts), bone resorption (osteoclasts), and the transduction of bone loading signals (osteocytes) (see Table 2). This conclusion is supported by the finding that our exercise program caused significant reductions in serum levels of CTXI, a reliable marker of bone resorption, and modest rises in osteocalcin, a reliable marker of bone formation [53]. Postexercise levels of CTXI correlated inversely with estradiol levels, suggesting that estradiol enhanced the antiresorptive effects of exercise, a conclusion in keeping with the report of others that the beneficial effect of exercise on bone density is blunted after menopause [54].

Our study participants averaged two and one-half hours of moderate intensity exercise per week: the same amount of exercise recommended for adult men and women by the World Health Organization for maintenance of health [55]. Participants divided their time between aerobic exercises (57%), resistance exercises (35%), and flexibility exercises (8%) and spent an average of 71 minutes in each training session. Extended over a six-month period, this amount of exercise was sufficient to reduce the spontaneous and PHA-induced production of osteoclastogenic cytokines by 24 and 59%, respectively, and to increase the spontaneous and PHA-induced production of antiosteoclastogenic cytokines by 89% and 50%, respectively. In PHA-stimulated cultures, osteoclastogenic cytokine production fell in proportion to the time subjects spent in each session doing aerobic exercises.

Cross-sectional studies involving adult subjects and using bone mineral density measurements have shown that exercises involving high impact (e.g., jumping) and high resistance (e.g., weightlifting) appear to be particularly effective in improving bone mass and content, especially when the intensity of the exercise is high and the speed of movement is elevated. Loaded (weight-bearing) aerobic exercises such as walking or running also have the potential to improve bone mass in adults and have the added benefit of improving cardiovascular conditioning [56, 57].

Studies on the impact of different exercise modalities on biomarkers of bone formation and resorption support the importance of both resistance and aerobic training in maintaining bone health. Fujimura and associates found that high intensity resistance training (weightlifting) done three times weekly for four months by 17 young male subjects caused a sustained increase in serum calcitonin levels, although plasma procollagen Type I C-terminal levels, a marker of bone resorption, did not change [58]. In a study involving 56 previously inactive young women, Lester and associates found that 8 weeks of resistance or combined aerobic-resistance training increased serum levels of osteocalcin but had no significant effect on markers of bone resorption [59]. In a study more closely resembling ours, Alghadir and associates found that 12 weeks of moderate intensity aerobic training done for 45 to 60 minutes three times weekly by 65 healthy subjects (36 males, 29 females) aged 30–60 increased serum osteocalcin levels and decreased serum levels of deoxypyridinoline, a marker of bone resorption [60].

In our study, it is of interest that postexercise culture levels of IL-6 were proportionate to weight, which is a measure of one's mass times the intensity of the gravity field (9.8 m/sec^2^ on Earth). It is possible, therefore, that the failure of exercise programs to attenuate bone loss in the microgravity of space is related, at least in part, to suboptimal production of this pleiotropic cytokine. IL-6 exerts context-dependent effects on bone metabolism [5, 30, 31]. Although it can stimulate osteoclastogenesis and bone resorption by upregulating RANKL expression in osteoblasts [32], its effects on bone metabolism are predominantly osteogenic and antiresorptive. IL-6 enhances bone formation by promoting the differentiation of osteoblast precursors [33, 34] and by protecting osteoblasts against apoptosis [5, 35]; it can inhibit bone resorption directly by downregulating RANKL signaling pathways in osteoclasts [36] and indirectly by suppressing the production of TNF-*α* and IL-1 and stimulating the production of IL-4, IL-10, and IL-1 receptor antagonist by immune cells [37, 38]. It is also an essential growth factor for B cells, the primary source of OPG in bone marrow stroma [39], and can induce IL-2 production in T cells [38, 40, 41]. IL-6 is produced in osteoblasts and osteocytes in response to bone loading signals and, like TGF-*β*, plays an important role in bone remodeling [42]. Thus, studies similar to that described in this report would be of particular interest if done on persons exercising in the microgravity of space.

This study would have benefitted from the inclusion of a nonexercising age- and sex-matched control group. Phenotypic analysis of Th17 and T regulatory cells and an assay for the mononuclear cell production of IL-17, a cytokine with osteoclastogenic activity, would also have been beneficial. Cardiorespiratory fitness measurements (VO_2max_) done before and after the exercise program was completed would have added potentially useful information.

## 5. Conclusions

Long-term moderate intensity exercise exerts a favorable effect on bone resorption by changing the balance between peripheral blood mononuclear cells producing osteoclastogenic cytokines and those producing antiosteoclastogenic cytokines. This beneficial effect may be enhanced by estradiol, emphasizing the importance of this hormone as a regulator of bone metabolism. The results provide a new insight as to how physical exercise contributes to the maintenance of bone health and suggest a possible molecular mechanism to explain the difference in the antiresorptive effects of exercise done on Earth as compared to exercise done in the microgravity of space.

## Figures and Tables

**Table 1:** Risk factors for osteoporosis^*∗*^.

	Men (*n* = 18)	Women (*n* = 25)
Age, mean (SD), y	48.1 (8.0)	49.7 (7.2)
Estradiol deficiency^a^	4 (28%)	15 (60%)
Inactivity^b^	11 (61%)	16 (64%)
Smoking	1 (6%)	4 (16%)
Alcohol^c^	9 (50%)	9 (36%)

^*∗*^Of the women, 68% were amenorrheic (44%, postmenopausal; 24%, posthysterectomy and oophorectomy). Of the amenorrheic women, 71% were taking estrogen replacement medication.

Eight percent of women had five risk factors, 12% had four risk factors, 24% had three risk factors, 36% had two risk factors, 16% had one risk factor, and 4% had no risk factors for osteoporosis. Fifty percent of men had one risk factor and 50% had two risk factors for osteoporosis. Twenty-four percent of women and 28% of men were obese (BMI > 30 kg/m^2^). None of the subjects were hyperthyroid or taking glucocorticoids.

^a^Normal values: men, 15–60 pg/mL; premenopausal women, 15–400 pg/mL (follicular phase, 15–120 pg/mL; ovulation, 200–400 pg/mL; luteal phase, 175–325 pg/mL); postmenopausal women, 0–40 pg/mL. ^b^No regular physical activity for ≥1 year. ^c^Five or more alcoholic beverages/week. Enrollment 25 (OH) vitamin D levels were normal (≥20 ng/mL) in all subjects.

**Table 2:** Modulation of bone and immune cells by cytokines.

Cytokine	Osteoblast	Osteoclast	Osteocyte	Bone (in vitro)	Rodents (in vivo)	T cells, B cells, and macrophages	References
IL1-*α*/*β*	↑ RANKL	↓ apoptosis		↑ resorption			[2–8, 15, 16]

TNF-*α*	↑ RANKL	↓ apoptosis ↑ RANKL-independent osteoclastogenesis		↑ resorption ↓ formation			[2–8, 16–18]

IFN-*γ*		↓ RANKL signaling pathways		↓ collagen synthesis	↑ bone loss ↓ osteopetrosis ↓ bone loss	↑ TNF-*α*, RANKL	[19–26]

IL-4	↓ RANKL ↑ OPG					↑ Th2-type ↓ Th1-type	[27–29]

Il-6	↑ RANKL ↑ precursors ↓ apoptosis	↓ RANKL signaling pathways	↑ production with loading			↓ TNF-*α*, IL-1*α*/*β* ↑ IL-4, IL-10, IL-1ra, OPG, and B cell maturation	[30–42]

Il-10	↑ OPG	↓ RANKL signaling pathways			↓ bone loss	↓ IFN-*γ*, IL-1*α*/*β*, TNF-*α*, and T helper cell proliferation	[43–46]

TGF-*β*	↓ RANKL, ↑ OB differentiation and synthesis of OPG and osteoid matrix	↓ osteoid degrading enzymes ↑ Wnt1	↑ production with loading	↑ osteoid matrix		↓ TNF-*α*, IL-1*α*/*β*, and IFN-*γ*	[41, 47–51]

RANKL, receptor activator of nuclear factor kappa B ligand, promoting osteoclastogenesis by binding to RANK on osteoclast precursors. OPG, osteoprotegerin; a decoy RANKL receptor & potent inhibitor of osteoclastogenesis. Wnt1, a protein crucial to normal bone formation. IL-1ra, interleukin-1 receptor antagonist.

## References

[1] Horton J. E., Raisz L. G., Simmons H. A., Oppenheim J. J., Mergenhagen S. E. (1972). Bone resorbing activity in supernatant fluid from cultured human peripheral blood leukocytes. *Science*.

[2] Dewhirst F. E., Stashenko P. P., Mole J. E., Tsurumachi T. (1985). Purification and partial sequence of human osteoclast-activating factor: identity with interleukin 1*β*. *Journal of Immunology*.

[3] Pfeilschifter J., Chenu C., Bird A., Mundy G. R., Roodman G. D. (1989). Interleukin-1 and tumor necrosis factor stimulate the formation of human osteoclast-like cells in vitro. *Journal of Bone and Mineral Research*.

[4] Ralston S. H., Gowen M. (1992). Role of cytokines in clinical disorders of bone metabolism. *Cytokines and Bone Metabolism*.

[5] Steeve K. T., Marc P., Sandrine T., Dominique H., Yannick F. (2004). IL-6, RANKL, TNF-alpha/IL-1: interrelations in bone resorption pathophysiology. *Cytokine and Growth Factor Reviews*.

[6] Mori G., D'Amelio P., Faccio R., Brunetti G. (2015). Bone-immune cell crosstalk: bone diseases. *Journal of Immunology Research*.

[7] Weitzmann M. N., Pacifici R. (2005). Role of the immune system in postmenopausal bone loss. *Current Osteoporosis Reports*.

[8] Wada T., Nakashima T., Hiroshi N., Penninger J. M. (2006). RANKL-RANK signaling in osteoclastogenesis and bone disease. *Trends in Molecular Medicine*.

[9] Zupan J., Jeras M., Marc J. (2013). Osteoimmunology and the influence of pro-inflammatory cytokines on osteoclasts. *Biochemia Medica*.

[10] Weitzmann M. N., Pacifici R. (2005). The role of T lymphocytes in bone metabolism. *Immunological Reviews*.

[11] US Department of Health and Human Services (2004). *Bone Health and Osteoporosis: A Report of the Surgeon General*.

[12] Henderson N. K., White C. P., Eisman J. A. (1998). The roles of exercise and fall risk reduction in the prevention of osteoporosis. *Endocrinology and Metabolism Clinics of North America*.

[13] Ulbrich C., Wehland M., Pietsch J. (2014). The impact of simulated and real microgravity on bone cells and mesenchymal stem cells. *BioMed Research International*.

[14] Smith S. M., Davis-Street J. E., Fesperman J. V. (2003). Evaluation of treadmill exercise in a lower body negative pressure chamber as a countermeasure for weightlessness-induced bone loss: a bed rest study with identical twins. *Journal of Bone and Mineral Research*.

[15] Dewhirst F. E., Ago J. M., Peros W. J., Stashenko P. (1987). Synergism between parathyroid hormone and interleukin 1 in stimulating bone resorption in organ culture. *Journal of Bone And Mineral Research*.

[16] Stashenko P., Dewhirst F. E., Peros W. J., Kent R. L., Ago J. M. (1987). Synergistic interactions between interleukin 1, tumor necrosis factor, and lymphotoxin in bone resorption. *Journal of Immunology*.

[17] Fuller K., Murphy C., Kirstein B., Fox S. W., Chambers T. J. (2002). TNF*α* potently activates osteoclasts, through a direct action independent of and strongly synergistic with RANKL. *Endocrinology*.

[18] Bertolini D. R., Nedwin G. E., Bringman T. S., Smith D. D., Mundy G. R. (1986). Stimulation of bone resorption and inhibition of bone formation in vitro by human tumour necrosis factors. *Nature*.

[19] Gao Y., Grassi F., Ryan M. R. (2007). IFN-*γ* stimulates osteoclast formation and bone loss in vivo via antigen-driven T cell activation. *Journal of Clinical Investigation*.

[20] Takayanagi H., Ogasawara K., Hida S. (2000). T-cell-mediated regulation of osteoclastogenesis by signalling cross-talk between RANKL and IFN-*γ*. *Nature*.

[21] Gowen M., Nedwin G. E., Mundy G. R. (1986). Preferential inhibition of cytokine-stimulated bone resorption of recombinant interferon gamma. *Journal of Bone and Mineral Research*.

[22] Duque G., Huang D. C., Dion N. (2011). Interferon-*λ* plays a role in bone formation in vivo and rescues osteoporosis in ovariectomized mice. *Journal of Bone and Mineral Research*.

[23] Smith D. D., Gowen M., Mundy G. R. (1987). Effects of interferon-*γ* and other cytokines on collagen synthesis in fetal rat bone cultures. *Endocrinology*.

[24] Beresford J. N., Taylor G. T., Triffitt J. T. (1990). Interferons and bone. A comparison of the effects of interferon-*α* and interferon-*γ* in cultures of human bone-derived cells and an osteosarcoma cell line. *European Journal of Biochemistry*.

[25] Mann G. N., Jacobs T. W., Buchinsky F. J. (1994). Interferon-*γ* causes loss of bone volume in vivo and fails to ameliorate cyclosporin A-induced osteopenia. *Endocrinology*.

[26] Key L. L., Rodriguiz R. M., Willi S. M. (1995). Long-term treatment of osteopetrosis with recombinant human interferon gamma. *The New England Journal of Medicine*.

[27] Moreno J. L., Kaczmarek M., Keegan A. D., Tondravi M. (2003). IL-4 suppresses osteoclast development and mature osteoclast function by a STAT6-dependent mechanism: irreversible inhibition of the differentiation program activated by RANKL. *Blood*.

[28] Yamada A., Takami M., Kawawa T. (2007). Interleukin-4 inhibition of osteoclast differentiation is stronger than that of interleukin-13 and they are equivalent for induction of osteoprotegerin production from osteoblasts. *Immunology*.

[29] Te Velde A. A., Huijbens R. J. F., Heije K., De Vries J. E., Figdor C. G. (1990). Interleukin-4 (IL-4) inhibits secretion of IL-1*β*, tumor necrosis factor *α*, and IL-6 by human monocytes. *Blood*.

[30] Blanchard F., Duplomb L., Baud'huin M., Brounais B. (2009). The dual role of IL-6-type cytokines on bone remodeling and bone tumors. *Cytokine and Growth Factor Reviews*.

[31] Franchimont N., Wertz S., Malaise M. (2005). Interleukin-6: an osteotropic factor influencing bone formation?. *Bone*.

[32] Palmqvist P., Persson E., Conaway H. H., Lerner U. H. (2002). IL-6, leukemia inhibitory factor, and oncostatin M stimulate bone resorption and regulate the expression of receptor activator of NF-*κ*B ligand, osteoprotegerin, and receptor activator of NF-*κ*B in mouse calvariae. *Journal of Immunology*.

[33] Erices A., Conget P., Rojas C., Minguell J. J. (2002). GP130 activation by soluble interleukin-6 receptor/interleukin-6 enhances osteoblastic differentiation of human bone marrow-derived mesenchymal stem cells. *Experimental Cell Research*.

[34] Itoh S., Udagawa N., Takahashi N. (2006). A critical role for interleukin-6 family-mediated Stat3 activation in osteoblast differentiation and bone formation. *Bone*.

[35] Jilka R. L., Weinstein R. S., Bellido T., Parfitt A. M., Manolagas S. C. (1998). Osteoblast programmed cell death (apoptosis): modulation by growth factors and cytokines. *Journal of Bone and Mineral Research*.

[36] Yoshitake F., Itoh S., Narita H., Ishihara K., Ebisu S. (2008). Interleukin-6 directly inhibits osteoclast differentiation by suppressing receptor activator of NF-*κ*B signaling pathways. *The Journal of Biological Chemistry*.

[37] Petersen A. M. W., Pedersen B. K. (2006). The role of IL-6 in mediating the anti-inflammatory effects of exercise. *Journal of Physiology and Pharmacology*.

[38] Hunter C. A., Jones S. A. (2015). IL-6 as a keystone cytokine in health and disease. *Nature Immunology*.

[39] Li Y., Toraldo G., Li A. (2007). B cells and T cells are critical for the preservation of bone homeostasis and attainment of peak bone mass in vivo. *Blood*.

[40] Löwik C. W. G. M., Gowen M. (1992). Differentiation inducing factors: leukemia inhibitory factor and interleukin-6. *Cytokines and Bone Metabolism*.

[41] Fukuno N., Matsui H., Kanda Y. (2011). TGF-*β*-activated kinase 1 mediates mechanical stress-induced IL-6 expression in osteoblasts. *Biochemical and Biophysical Research Communications*.

[42] Bakker A. D., Kulkarni R. N., Klein-Nulend J., Lems W. F. (2014). IL-6 alters osteocyte signaling toward osteoblasts but not osteoclasts. *Journal of Dental Research*.

[43] Evans K. E., Fox S. W. (2007). Interleukin-10 inhibits osteoclastogenesis by reducing NFATc1 expression and preventing its translocation to the nucleus. *BMC Cell Biology*.

[44] Fiorentino D. F., Zlotnik A., Mosmann T. R., Howard M., O'Garra A. (1991). IL-10 inhibits cytokine production by activated macrophages. *The Journal of Immunology*.

[45] D'Andrea A., Aste-Amezaga M., Valiante N. M., Ma X., Kubin M., Trinchieri G. (1993). Interleukin 10 (IL-10) inhibits human lymphocyte interferon *γ*-production by suppressing natural killer cell stimulatory factor/IL-12 synthesis in accessory cells. *Journal of Experimental Medicine*.

[46] Dresner-Pollak R., Gelb N., Rachmilewitz D., Karmeli F., Weinreb M. (2004). Interleukin 10-deficient mice develop osteopenia, decreased bone formation, and mechanical fragility of long bones. *Gastroenterology*.

[47] Mundy G. R., Bonewald L. F., Gowen M. (1992). Transforming growth factor beta. *Cytokines and Bone Metabolism*.

[48] Quinn J. M. W., Itoh K., Udagawa N. (2001). Transforming growth factor *β* affects osteoclast differentiation via direct and indirect actions. *Journal of Bone and Mineral Research*.

[49] Schwartz Z., Olivares-Navarrete R., Wieland M., Cochran D. L., Boyan B. D. (2009). Mechanisms regulating increased production of osteoprotegerin by osteoblasts cultured on microstructured titanium surfaces. *Biomaterials*.

[50] Kini U., Nandeesh B. N., Fogelman I., Gnanasegaran G., van der Wall H. (2012). Physiology of bone formation, remodeling, and metabolism. *Radionuclide and Hybrid Bone Imaging*.

[51] Weivoda M. M., Ruan M., Pederson L. (2016). Osteoclast TGF-*β* receptor signaling induces Wnt1 secretion and couples bone resorption to bone formation. *Journal of Bone and Mineral Research*.

[52] Smith J. K., Dykes R., Douglas J. E., Krishnaswamy G., Berk S. (1999). Long-term exercise and atherogenic activity of blood mononuclear cells in persons at risk of developing ischemic heart disease. *The Journal of the American Medical Association*.

[53] Garnero P., Delmas P. D. (1998). Biochemical markers of bone turnover: Applications for osteoporosis. *Endocrinology and Metabolism Clinics of North America*.

[54] Bassey E. J., Rothwell M. C., Littlewood J. J., Pye D. W. (1998). Pre- and postmenopausal women have different bone mineral density responses to the same high-impact exercise. *Journal of Bone and Mineral Research*.

[55] World Health Organization (2014). *Global Status Report on Noncommunicable Diseases*.

[56] Guadalupe-Grau A., Fuentes T., Guerra B., Calbet J. A. L. (2009). Exercise and bone mass in adults. *Sports Medicine*.

[57] Gómez-Cabello A., Ara I., González-Agüero A., Casajús J. A., Vicente-Rodríguez G. (2012). Effects of training on bone mass in older adults: a systematic review. *Sports Medicine*.

[58] Fujimura R., Ashizawa N., Watanabe M. (1997). Effect of resistance exercise training on bone formation and resorption in young male subjects assessed by biomarkers of bone metabolism. *Journal of Bone and Mineral Research*.

[59] Lester M. E., Urso M. L., Evans R. K. (2009). Influence of exercise mode and osteogenic index on bone biomarker responses during short-term physical training. *Bone*.

[60] Alghadir A. H., Aly F. A., Gabr S. A. (2014). Effect of moderate aerobic training on bone metabolism indices among adult humans. *Pakistan Journal of Medical Sciences*.

